# Recombinant Attenuated *Salmonella* Enteritidis Vector Enhances the Immunogenicity of *Clostridium perfringens* EntB Antigen for Effective Prevention of Avian Necrotic Enteritis

**DOI:** 10.3390/biom16040575

**Published:** 2026-04-13

**Authors:** Wenjing Li, Yu-An Li, Xiaolong Liu, Haiping Xie, Jingyi Zhao, Yi Feng, Huoying Shi

**Affiliations:** 1 College of Veterinary Medicine, Yangzhou University, Yangzhou 225009, China; dz120220033@stu.yzu.edu.cn (W.L.); 008330@yzu.edu.cn (Y.-A.L.); mx120230953@stu.yzu.edu.cn (X.L.); dz120250051@stu.yzu.edu.cn (H.X.); mz120231742@stu.yzu.edu.cn (J.Z.); mx120230955@stu.yzu.edu.cn (Y.F.); 2 Jiangsu Co-Innovation Center for the Prevention and Control of Important Animal Infectious Diseases and Zoonoses, Yangzhou 225009, China; 3 Joint International Research Laboratory of Agriculture and Agri-Product Safety, Yangzhou University, Yangzhou 225009, China

**Keywords:** necrotic enteritis, *Salmonella* Enteritidis vector, immunogenicity, EntB protein, vaccine development

## Abstract

Necrotizing enteritis (NE) is an important intestinal disease threatening the poultry farming industry, and the ban on antibiotic growth promoters has created an urgent demand for safe and effective NE vaccines. Recombinant attenuated *Salmonella* vectors (RASVs) administered orally can induce mucosal immune responses against delivered antigens, thus showing great potential to elicit protective immunity against NE. The EntB protein is a newly discovered putative enterotoxin of *Clostridium perfringens* (*C. perfringens*). Bioinformatic predictions in this study revealed that EntB contains nineteen potential antigenic epitopes, two functional domains (NlpC and YgiM), and interacts with ten proteins, supporting its potential as a target antigen for NE vaccines. To optimize the immunogenicity of EntB-based vaccines, we constructed a novel recombinant attenuated *Salmonella* Enteritidis (*S.* Enteritidis) vector rSC0169 harboring a rhamnose-regulated delayed attenuation system, which was then used to deliver EntB to generate the recombinant strain rSC0169(pS-EntB). This system enhanced the immunogenicity of the *Salmonella* vector rSC0169 and further elicited robust mucosal immune responses against EntB, as well as humoral and cellular immune responses. Compared with the control strain rSC0169(pS0018), rSC0169(pS-EntB) candidate vaccine strain significantly alleviated NE symptoms, increased the intestinal villus height/crypt depth (VH/CD) ratio, upregulated tight junction protein expression, and reduced excessive pro-inflammatory cytokine production. In conclusion, this study provides a promising NE candidate vaccine and offers a valuable strategy for developing vaccines against other intestinal bacterial diseases.

## 1. Introduction

Necrotizing enteritis (NE), caused by *Clostridium perfringens* (*C. perfringens*), is a severe intestinal bacterial disease affecting the global poultry industry, with annual economic losses of approximately 6 billion US dollars. Notably, poultry products contaminated with *C. perfringens* also trigger foodborne poisoning, posing a global public health challenge [1]. Antibiotics have been widely added to animal feed as growth promoters to prevent diseases. However, the extensive use of antibiotics has led to the emergence of drug-resistant strains, which is a serious public health problem [2]. Vaccination is the most effective approach for preventing and controlling infectious diseases. Several *C. perfringens* vaccine candidates have been tested, but most exhibit poor ability to elicit mucosal immune responses, fail to confer protection, or pose biosafety risks [3]. Therefore, developing an effective NE vaccine remains a significant challenge.

The exotoxins of *C. perfringens* are key triggers of necrotic enteritis and important targets for vaccine development [4,5]. Among these toxins, NetB is the most extensively characterized key virulence factor and has been widely employed as the primary antigen in NE vaccine design. In contrast, EntB is a novel and distinct immunogenic antigen identified from type G strains [1]. As a secreted putative enterotoxin, EntB is proposed to act through a different pathogenic mechanism and may confer complementary protection to that provided by NetB-based vaccines. Although EntB has been preliminarily demonstrated to have immunogenicity, it is unable to induce mucosal immune responses. Furthermore, little is known regarding its protective efficacy against *C. perfringens*, particularly regarding its performance when delivered by specific vectors/adjuvant platforms.

Attenuated *Salmonella* vectors induce a robust mucosal immune response, which is crucial for combating intestinal pathogens, such as NE [6]. However, attenuated *Salmonella* vectors pose a biosafety risk of environmental shedding from immunized hosts, and excessive attenuation can impair immunogenicity and ultimately cause vaccine failure [7]. To overcome these drawbacks, we previously constructed the *S.* Enteritidis vector rSC0163 containing the *Salmonella* mRNA interferase regulation vector (SIRV) system, which can undergo regulated lysis to release cytoplasmic DNA and activate the cGAS-STING innate immune pathway [8]. Nevertheless, the *S.* Enteritidis vector rSC0163 still requires further optimization to improve its biosafety profile and enhance immune responses against delivered heterologous antigens.

To achieve this goal, we engineered an improved vector strain rSC0169 by introducing a combination of mutations: Δ*sifA*, a rhamnose-regulated delayed attenuation system, Δ*recF*, and Δ*asdA*. The Δ*sifA* mutation causes the exogenous antigens carried by *Salmonella* to be released into the cytoplasm of immune cells, which can significantly enhance the immune response triggered by the exogenous antigens [9]. A rhamnose-regulated delayed attenuation system enhances vector immunogenicity and biosafety, and consequently elevates the immunogenicity of the delivered antigens [10]. The system enables *Salmonella* vectors to effectively colonize deep lymphoid tissues at the initial stage of immunity, which helps enhance vector immunogenicity. Subsequently, it exhibits attenuation characteristics in rhamnose-free hosts, thereby ensuring sufficient vaccine biosafety. The recombination of plasmids in *Salmonella* can lead to the loss or dysfunction of gene fragments, directly affecting the efficacy of the vaccine. The Δ*recF* mutation can enhance the stability of the plasmid delivered by the *Salmonella* vector [11,12]. In addition, the Δ*asdA* mutation establishes a balanced-lethal system that facilitates the stable application of *Salmonella* as an antigen delivery carrier without antibiotic selection [13]. Overall, these mutations act synergistically to enhance antigen presentation, plasmid stability, and vector safety, thereby establishing a solid foundation for efficient delivery of heterologous antigens using the rSC0169 strain.

In this study, we introduced the Δ*sifA*, the rhamnose-regulated delayed attenuation system, Δ*recF*, and Δ*asdA* into the *Salmonella* vector rSC0163 to construct a new recombinant attenuated *Salmonella* vector, rSC0169, to improve its immunogenicity and biosafety. Subsequently, the *Salmonella* vector rSC0169 was used to deliver the new antigen EntB for constructing the vaccine candidate rSC0169(pS-EntB). The immunogenicity, safety, and protective efficacy of rSC0169(pS-EntB) against NE were evaluated, aiming to develop a safe and efficient NE vaccine.

## 2. Materials and Methods

### 2.1. Animals and Ethics Statement

One-day-old male Ross 308 broilers were procured from a commercial farm (Jiangsu, China). Animal experiments were approved by the Laboratory Animal Ethics Committee of Yangzhou University (approval no. 202403651) and complied with the regulations of the Jiangsu Administrative Committee for Laboratory Animals.

### 2.2. Plasmids, Strains, and Growth Conditions

The ACA- plasmid pS0018, derived from plasmid pYA3493, lacks the ACA nucleotide sequence without changing the encoded amino acid sequence of the *asdA* gene [14]. The ACA- plasmid pS-EntB, derived from pS0018, carries the *entB* gene (ACA sequence deletion does not alter the encoded amino acid sequence). *Escherichia coli* (*E. coli*) χ7213 carrying plasmids pS0018 (empty vector) and pS-EntB (expression vector) was cultured in Luria broth (LB; Oxoid, Basingstoke, UK) or LB agar medium. Recombinant *S.* Enteritidis vector rSC0169 delivering these plasmids was cultured in LB broth supplemented with 0.2% L-arabinose (*w*/*v*), 0.1% L-rhamnose (*w*/*v*), and 0.2% D-mannose (*w*/*v*) (Sigma-Aldrich, St. Louis, MO, USA). The *C. perfringens* strain rSC0097 used for experimental infection was isolated from a chicken suffering from necrotizing enteritis. This pathogenic strain belongs to type G and is positive for α-toxin and NetB toxin but negative for TpeL. Prior to bacterial challenge, *C. perfringens* strain rSC0097 was revived on sheep blood agar. Ten single colonies were inoculated into cooked meat medium (CMM; Hopebio, Qingdao, China), incubated anaerobically for 12 h, then inoculated at 3% (*v*/*v*) into fluid thioglycolate medium (FTG; Hopebio, Qingdao, China) supplemented with 5% beef extract (Solarbio, Beijing, China), and grown at 37 °C for 15 h to prepare the challenge inoculum [1,10]. The growth curves of rSC0169(pS0018) and rSC0169(pS-EntB) were determined by measuring optical density (OD_600_). Overnight bacterial cultures were adjusted to a uniformly diluted to an initial OD_600_, and then transferred to 5 mL of LB medium (0.2% L-arabinose, 0.1% L-rhamnose, and 0.2% D-mannose) at a ratio of 1:100. OD_600_ was measured every 2 h for a total of 12 h to generate the growth curves [15]. Plasmids and strains employed are summarized in Table 1.

### 2.3. Sequence Analysis of EntB

To further characterize the immunogenicity and structural properties of EntB, the potential antigenic epitopes were predicted via http://imed.med.ucm.es/Tools/antigenic.pl (accessed on 5 November 2025). Meanwhile, its tertiary structure, conserved domains and protein–protein interaction network were, respectively, predicted via Swiss-Model (https://swissmodel.expasy.org/interactive, accessed on 5 November 2025), the NCBI website (http://www.ncbi.nlm.nih.gov/, accessed on 5 November 2025) and the STRING database (https://cn.string-db.org/, accessed on 5 November 2025) [16].

### 2.4. Construction of S. Enteritidis Vector rSC0169

The *Salmonella* vector was engineered according to previously described methods [8]. Mutations including Δ*sifA*, Δ*waaL*, Δ*pagL64*::TT *rhaRs*P_rhaBAD_ *waaL*, Δ*recF*, and Δ*asdA* were sequentially introduced into *S.* Enteritidis vector rSC0163 via standard homologous recombination to generate vector rSC0169. For culturing the donor strains, 0.2% L-arabinose was added, whereas for suicide vector recipient bacteria, DAP (Sigma-Aldrich, St. Louis, MO, USA) was added. Fifty microliters each of donor and recipient bacterial cultures were spotted onto LB plates (0.2% L-arabinose and DAP) and incubated overnight. Bacterial lawn was streaked onto LB plates with 0.2% L-arabinose and 50 µg/mL *Cm* (Sangon, Shanghai, China) for cultivation. Eight single colonies were picked and purified on LB plates (0.2% L-arabinose and 50 µg/mL *Cm*). After purification, the single colonies were cultured in LB broth, then serially diluted to an appropriate concentration and spread onto LB plates with 0.2% L-arabinose and 5% sucrose (Sigma-Aldrich, St. Louis, MO, USA). During *asd* gene deletion in the recipient bacteria, the purified single-colony cultures were spread onto LB plates containing 0.2% L-arabinose, 5% sucrose, and DAP. Finally, single colonies without chloramphenicol resistance were verified by PCR using the primers listed in Table 2. Notably, Δ*waaL* and Δ*pagL64*::TT *rhaRs*P_rhaBAD_ *waaL* mutations jointly form a rhamnose-regulated delayed attenuation system.

### 2.5. Characterization of Regulatory Elements in Rhamnose-Regulated Delayed Attenuation System

The immunogenicity and safety evaluation procedures were performed with minor modifications based on a previously described method [17]. One-day-old chicks were randomized into 4 groups (*n* = 3 per group) by simple randomization and orally inoculated with 1 ± 0.2 × 10^9^ CFU (200 µL) of *S.* Enteritidis C50041, rSC0169 (cultured with or without rhamnose), or PBS. Two weeks later, a booster vaccination with the same dose was administered to each group. On day 7 post-booster vaccination, serum, intestinal mucosal rinse, spleens, and livers were harvested from the chickens [18]. Enzyme-linked immunosorbent assay (ELISA) was used to detect the antibody levels against the outer membrane proteins (OMPs) of *S.* Enteritidis C50041 in serum and intestinal mucosal rinse. Quantitative real-time PCR (qRT-PCR) was performed to quantify the IL-1β and IL-18 mRNA levels in the spleen. The results were normalized to chicken β-actin mRNA expression, and the corresponding primer sequences are listed in Table 2. Liver tissues were embedded in paraffin, sectioned, and stained with hematoxylin and eosin (H&E; Zhuhai, China) for lesion observation.

### 2.6. Preparation of OMPs

The OMPs of *S.* Enteritidis C50041 strain were prepared according to a previously described method [8]. Briefly, the wild-type *S.* Enteritidis C50041 strain was cultivated in 50 mL LB medium to mid-log phase (OD_600_ ≈ 0.8). The bacterial culture was harvested by centrifugation and resuspended in 5 mL of 100 mM Tris-HCl buffer (pH 8.6; Biosharp, Hefei, China) containing 0.5 mM EDTA (Sigma-Aldrich, St. Louis, MO, USA). Subsequently, 40 μL of 40 mg/mL egg white lysozyme (Beyotime, Shanghai, China) was added, followed by 3.2 mL of 50 mM Tris-HCl (pH 8.6) containing 25 mM MgCl_2_ (Beyotime, Shanghai, China). The mixture was gently shaken and incubated on ice for 15 min. Bacterial cells were lysed by sonication using a Sonics Vibra Cell apparatus (Sonics & Materials, Inc., Newtown, CT, USA). The lysate was centrifuged at 4 °C for 6 min at 7000× *g*, and the resulting supernatant was collected and further centrifuged at 4 °C for 1 h at 13,200× *g* to pellet the crude membrane fraction. The precipitate was resuspended in 4 mL of 20 mM Tris-HCl (pH 8.6) containing 1% sodium N-lauroylsarcosinate (Sigma-Aldrich, St. Louis, MO, USA) and incubated on ice for 30 min. The suspension was then centrifuged at 4 °C for 1 h at 13,200× *g*, and the resulting precipitate was resuspended in 4 mL of 20 mM Tris-HCl (pH 8.6) to obtain the outer membrane proteins.

### 2.7. Macrophage Infection Assay

The infection experiments of macrophages were conducted using a previously published procedure [8]. RAW264.7 cells (1.0 × 10^6^ cells per well) were infected with PBS (blank control), C50041 (positive control), or rSC0169 (cultured with or without rhamnose), at a multiplicity of infection of 10 (MOI). At 12 h post-infection, cells were collected and lysed in cell lysis buffer. Samples were separated by SDS-PAGE, followed by Western blot analysis using primary antibodies against IRF3 and phospho-IRF3 (Cell Signaling Technology, Danvers, MA, USA), with β-actin (Abcam, Cambridge, UK) as the internal reference.

### 2.8. Construction and Genetic Stability of rSC0169(pS-EntB)

To generate the recombinant vaccine candidates, plasmid pS-EntB was transformed into electrocompetent asd-deficient *S.* Enteritidis vector rSC0169. The resulting transformants were selected and purified on LB agar plates (0.2% L-arabinose). PCR verification was performed using specific primers for pS0018. To assess genetic stability, rSC0169(pS-EntB) was serially passaged in LB medium (0.2% L-arabinose, 0.1% L-rhamnose, and 0.2% D-mannose) at a ratio of 1:100, with each generation incubated for 12 h, for a total of 50 generations. Samples were collected every 10 generations and verified by PCR with the same specific primers for pS0018 to confirm genetic stability [15].

### 2.9. S. Enteritidis Subcellular Fractionation

To assess the subcellular localization of EntB synthesized in rSC0169(pS-EntB), culture supernatant, periplasmic, and cytoplasmic fractions were extracted. Periplasmic fractions were prepared using a modified lysozyme-osmotic shock method [15]. Briefly, rSC0169(pS-EntB) was grown in LB medium to an OD_600_ of 0.8 and then induced with 0.5 mM isopropyl-β-D-thiogalactoside (IPTG; Sangon, Shanghai, China) for 3 h. The 50 mL bacterial culture supernatant was filtered, precipitated with 10% trichloroacetic acid (4 °C, 1 h), centrifuged (16,000× *g*, 4 °C, 1 h), washed with acetone stock solution, and resuspended in 4 mL of 20 mM Tris-HCl (pH 8.6). Cell pellets were first resuspended in 800 μL 100 mM Tris-HCl buffer containing 500 mM sucrose and 0.5 mM EDTA. Next, 40 μL of lysozyme (4 mg/mL) was added to the suspension, followed by 3.2 mL of 50 mM Tris-HCl buffer containing 250 mM sucrose, 0.25 mM EDTA, and 2.5 mM MgCl_2_. The mixture was agitated, chilled on ice for 15 min, centrifuged at 7000× *g* for 6 min, and the supernatant was filtered to collect the periplasmic fraction. Finally, the remaining cells were resuspended in 4 mL of 20 mM Tris-HCl, disrupted via ultrasonication, and the lysate was collected. Equal volumes of periplasmic, cytoplasmic, and supernatant fractions were separated by 10% or 15% SDS-PAGE and analyzed by Western blot using mouse polyclonal antibodies against EntB, PlcC, and NetB (1:1000).

### 2.10. Distribution of rSC0169(pS-EntB) in Chickens

To analyze the distribution of *Salmonella*, one-day-old chicks were randomized into two groups (*n* = 12 per group) by simple randomization and orally inoculated with nalidixic acid-resistant mutants rSC0169(pS0018) and rSC0169(pS-EntB) at 1 × 10^9^ CFU per chicken. On 3, 7, 14, and 21 days post-inoculation (dpi), three chickens per group were randomly selected, euthanized, and sampled for liver, spleen, and cecal lymph node tissues. These tissues were weighed and then homogenized with PBS. Bacterial loads in these tissues were quantified using established methods, with CFUs counted on MacConkey agar plates (Hopebio, Qingdao, China) containing 0.2% L-arabinose, 1% D-lactose, and 100 μg/mL nalidixic acid (Solarbio, Beijing, China) [8].

### 2.11. Immunization and Challenge

To assess the immune protective efficacy of vaccine candidates, one-day-old chicks were randomized into 4 groups (*n* = 7 per group) by simple randomization: blank control group, challenge group, empty vector group rSC0169(pS0018), and rSC0169(pS-EntB) immune group. Chickens in the rSC0169(pS0018) and rSC0169(pS-EntB) groups received oral administration of the corresponding *S.* Enteritidis strains (1 × 10^9^ CFU/200 μL), whereas the blank control group was gavaged with PBS (200 μL). Booster immunization with the same dose was administered 2 weeks post-primary immunization (i.e., day 14).

A modified *Eimeria maxima* (*E. maxima*)-induced NE challenge model was established and employed in this study [1,10]. *E. maxima* oocysts were allowed to stand at room temperature for at least 2 h prior to oral inoculation. All groups except the blank control group were orally inoculated with 3000 fresh *E. maxima* oocysts at 23 days of age. Feed and water were withheld 24 h before coccidiosis inoculation. Subsequently, feed was restored 2 h before inoculation, and water was restored 2 h after inoculation. From days 28 to 30, all groups except the blank control group received 1 mL of fresh *C. perfringens* culture (at a concentration of approximately 5 × 10^8^ CFU/mL). Fresh broth cultures were prepared daily. No fasting was performed before oral gavage with *C. perfringens*.

### 2.12. ELISA

Recombinant EntB protein expressed by BL21-pET28a(+)-EntB was purified using Nichelating affinity gel (Genscript, Nanjing, China). Serum and intestinal mucosal rinse samples were collected 1 week post-booster immunization, and the titers of EntB-specific IgY and IgA antibodies were measured via ELISA [1]. Briefly, 96-well polystyrene plates were coated with 100 μL of EntB (1 μg/well) diluted in coating buffer (0.1 M carbonate buffer, pH 9.6) and incubated overnight at 4 °C. After washing with PBST (PBS containing 0.5% Tween 20), the plates were blocked with 5% non-fat milk powder in PBST for 2 h at 37 °C. Following another wash, diluted serum samples (1:50) and intestinal mucosal rinse samples (1:5) prepared in PBST were added to the corresponding wells and incubated for 2 h at 37 °C. After washing, the plates were incubated with 100 μL of goat anti-chicken IgY-HRP (1:5000, SouthernBiotech, Birmingham, AL, USA) or goat anti-chicken IgA-HRP (1:5000, Abcam, Cambridge, UK) for 1 h at 37 °C. After further washing, 100 μL of 3, 3′, 5, 5′-Tetramethylbenzidine (TMB; Solarbio, Beijing, China) substrate solution was added to each well, and the reaction was developed for 15 min at 37 °C. The reaction was terminated by adding 50 μL of 2 M H_2_SO_4_, and the optical density (OD) value was measured at 450 nm.

### 2.13. Cytokine Detection

The mRNA expression levels of IFN-γ, IL-4, and IL-17A in EntB-stimulated peripheral blood mononuclear cells (PBMCs) were quantified using qRT-PCR [1]. Briefly, PBMCs were isolated from vaccinated chickens at 1 week post-vaccination using a chicken PBMC isolation kit (Solarbio, Beijing, China). After resuspending PBMCs in RPMI 1640 medium (Hyclone, Logan, UT, USA) supplemented with 10% FBS (Gibco, Waltham, MA, USA) and 1% penicillin/streptomycin (Sangon, Shanghai, China), cells were seeded into 24-well plates (1 × 10^7^ cells per well) and incubated the plates at 37 °C and 5% CO_2_ for 48 h. The cells were stimulated separately with 20 μg/mL of EntB, PlcC, or NetB, or with 5 μg/mL Concanavalin A (ConA; Solarbio, Beijing, China) as a positive control. Cell samples were collected for total RNA extraction (Vazyme, Nanjing, China), followed by cDNA synthesis via reverse transcription to quantify cytokine mRNA levels. Primers used are listed in Table 2. The mRNA expression levels of cytokines were analyzed using the 2^−ΔΔCt^ method.

### 2.14. Lymphocyte Proliferation Assays

Lymphocyte proliferation assays were assessed using the CCK-8 assay as previously described [1]. One week after the booster vaccination, three chickens per group were randomly selected for blood collection to isolate PBMCs. Briefly, PBMCs were stimulated as described above (2 × 10^5^ cells/well), followed by addition of 10 µL CCK-8 reagent (Solarbio, Beijing, China). After 4 h of incubation, the OD_450_ values were measured to assess lymphocyte proliferation. The stimulation index (SI) was calculated as the ratio of the OD_450_ of antigen-stimulated wells to those of unstimulated cells.

### 2.15. Necrotic Lesion Score and Histopathological Score

Chickens were euthanized and dissected at 4 h post-*C. perfringens* challenge. NE lesion severity was assessed using a 0–6 scoring system [1,10]. Small intestinal tissues were fixed, embedded in paraffin, sectioned, and stained with H&E for histopathological examination. Histopathological lesion scoring was conducted as previously described [1]. All lesion and histopathological scoring was performed in a blinded manner.

### 2.16. Small Intestine Morphology

Small intestinal morphology from the same anatomical region of each chicken was observed as reported previously [19]. After H&E staining, five intact villi per tissue section were randomly selected for measurement. Villus height was measured as the distance from villus tip to the crypt mouth, and crypt depth as the distance from the villus base to submucosa. The villus height-to-crypt depth ratio (VH/CD) was calculated, with the mean value of five measurements per chicken used for statistical analysis.

### 2.17. Small Intestine Gene Expression

Total RNA was isolated from small intestinal tissues per the manufacturer’s instructions, reverse transcribed to cDNA, and analyzed by qRT-PCR for ZO-1, CLDN-1, IL-1β, and IL-6 expression [20,21]. Relative gene expression levels were calculated via the 2^−ΔΔCt^ method, with β-actin as the reference gene. The primer sequences are presented in Table 2.

### 2.18. Statistical Analysis

Statistical analyses were conducted using GraphPad Prism software (version 10.1.2). Data normality was assessed for all variables prior to analysis. Based on the number of comparison groups, the Mann–Whitney U test was used for two-group comparisons, and the Kruskal–Wallis test was employed for comparisons among multiple groups. Data are presented as mean ± SD, and differences were considered statistically significant at *p* < 0.05. All experiments were performed in triplicate. Data analysis was performed in a blinded manner to minimize observer bias.

## 3. Results

### 3.1. Sequence Analysis of EntB Gene

**Figure 1:**
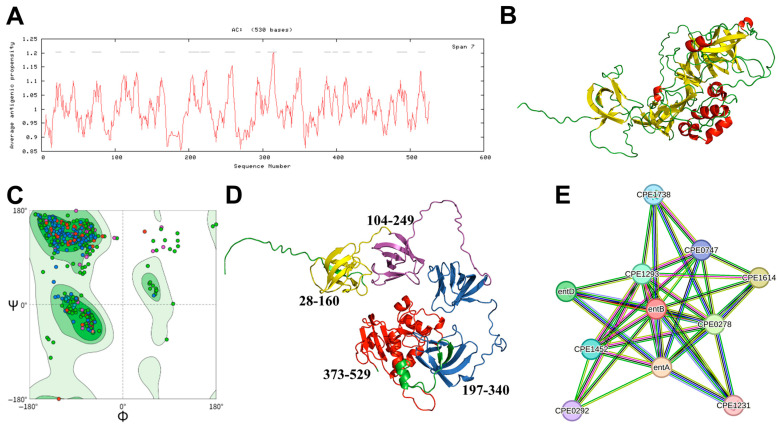
Characteristics of the EntB protein. (**A**) Antigenic determinant prediction. (**B**) Tertiary structure prediction. (**C**) Ramachandran plot of the EntB tertiary structure. Colored dots represent individual amino acid residues. The dark green shaded region indicates the most favored region, with 90.51% of amino acids located in the favored region. (**D**) Domain prediction. (**E**) Protein–protein interaction network of EntB constructed using the STRING database. All bioinformatic predictions were performed using online tools as described in Section 2.

Obtaining information regarding protein antigenic epitopes and functional domains is critical for rational vaccine design [22]. Toxin antigens represent ideal targets for NE vaccine development. EntB is a newly identified potential enterotoxin [1]. Epitope prediction analysis revealed that EntB contains 19 potential antigenic epitopes, with an average antigen tendency index of 1.0237 (Figure 1A). The tertiary structure of EntB was visualized using SWISS-MODEL, where α-helices, β-sheets, and random coils were colored red, yellow, and green, respectively (Figure 1B). Ramachandran plot analysis verified structural reliability, with 90.51% of amino acids localized in favorable regions (Figure 1C). Identifying functional domains of proteins is crucial for vaccine development and investigation of host immune responses. The EntB protein contains two domains: an NlpC domain (registration number: COG0791) spanning 373–529 amino acid residues (red area), and a YgiM domain (registration number: COG3103) distributed across residues 28–160 (yellow area), 104–249 (purple area) and 197–340 (blue area) (Figure 1D). Furthermore, protein–protein interaction analysis indicated that EntB interacts with 10 proteins, implying its potential involvement in multiple biological processes (Figure 1E). Collectively, these data provide a theoretical basis for developing EntB-based candidate vaccines against NE.

### 3.2. Construction of Rhamnose-Regulated S. Enteritidis Vector rSC0169 and Vaccine Candidate Strain rSC0169(pS-EntB)

To improve the safety and immunogenicity of the *S.* Enteritidis vector strain, five mutations (Δ*sifA*, Δ*waaL*, Δ*pagL64*::TT *rhaRs*P_rhaBAD_ *waaL*, Δ*recF*, and Δ*asdA*) were successfully introduced into the parental vector strain rSC0163, generating a new recombinant attenuated *S.* Enteritidis vector rSC0169.

### 3.3. Rhamnose-Regulated S. Enteritidis Vector rSC0169 Exhibits Favorable Safety in Chickens

**Figure 2:**
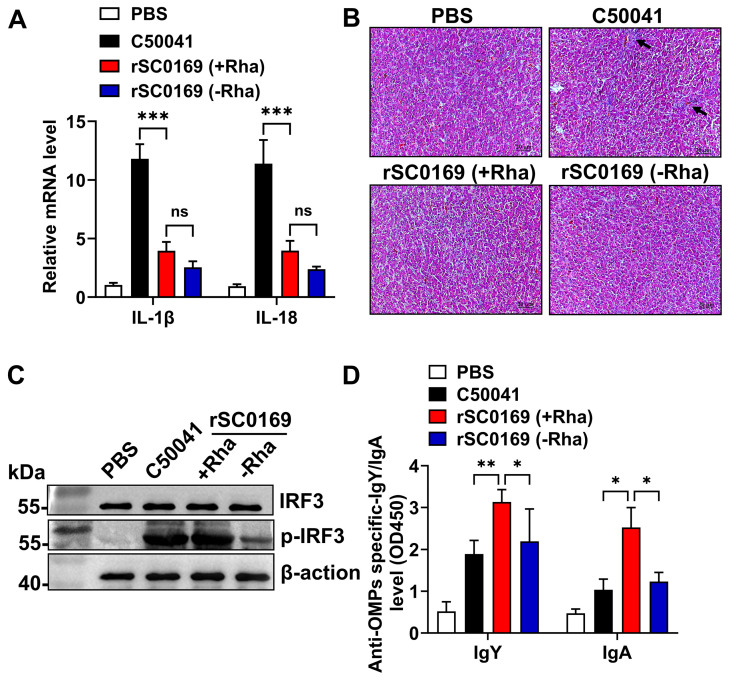
Immunogenicity and safety analysis of the rhamnose-regulated *S.* Enteritidis vector rSC0169. (**A**,**B**) Transcriptional levels of pro-inflammatory cytokines IL-1β and IL-18 in chicken spleen (**A**) and liver inflammatory response (**B**) at 7 days post-booster immunization. Data are shown as mean ± SD (*n* = 3). Scale bar: 20 μm. Black arrows indicate inflammatory cell infiltration. (**C**) IRF3 and p-IRF3 expression in RAW264.7 cells infected with the indicated *S.* Enteritidis strains. PBS: negative control; β-actin: loading control. (**D**) Specific anti-OMPs IgY and IgA titers in chicken serum and intestinal mucosal rinse determined by ELISA. Abbreviations: +Rha, strain cultured with rhamnose; -Rha, strain cultured without rhamnose. ns means not significant. *, *p* < 0.05, **, *p* < 0.01, ***, *p* < 0.001. Original images of (**C**) can be found in Supplementary Materials.

Excessive inflammatory responses promote disease development in avian hosts [23]. To evaluate the inflammatory response of *S.* Enteritidis strains in avian hosts, spleens and livers were harvested from immunized chickens at 7 days post-booster immunization. Chickens inoculated with rSC0169 (cultured with or without rhamnose) exhibited significantly reduced IL-1β (0.33-fold and 0.22-fold relative to C50041; *p* < 0.001) and IL-18 (0.35-fold and 0.21-fold relative to C50041; *p* < 0.001) transcript levels in the spleen compared to those inoculated with wild-type *S.* Enteritidis C50041 (Figure 2A). Consistently, liver histopathology showed no lesions in the PBS control group, while obvious inflammatory cell infiltration (black arrows) was observed in the *S.* Enteritidis C50041-inoculated group. In contrast, liver pathological lesions were significantly alleviated in chickens inoculated with rSC0169 (cultured with or without rhamnose) (Figure 2B). These results indicate that the rSC0169 with the rhamnose-regulated delayed attenuation system does not elicit excessive inflammatory responses in chickens, validating its biosafety as a vaccine vector.

### 3.4. Rhamnose-Regulated S. Enteritidis Vector rSC0169 Promotes a Higher Level of IRF3 Phosphorylation in RAW264.7 Cells

IFN regulatory factor 3 (IRF3) is a key transcription factor that modulates cellular responses and is indispensable for the initiation of innate immunity [24]. We detected the levels of total IRF3 and its phosphorylated active form (p-IRF3) in RAW264.7 cells following infection. Relative to the rSC0169 (cultured without rhamnose), infection with either the wild-type *S.* Enteritidis C50041 or the rSC0169 (cultured with rhamnose) significantly enhanced IRF3 phosphorylation in RAW264.7 cells (Figure 2C). No p-IRF3 activation was detected in PBS-treated cells. These data indicate that the rSC0169 (cultured with rhamnose) is functionally similar to the wild-type *S.* Enteritidis C50041, and thus can effectively trigger the IRF3-mediated innate immune signaling pathway in vitro.

### 3.5. Rhamnose-Regulated Delayed Attenuation System Enhances the Immunogenicity of S. Enteritidis Vector rSC0169

The rhamnose-regulated delayed attenuation system can enhance the immunogenicity of vectors [10]. To evaluate the effect of this system on the immunogenicity of rSC0169, we detected the specific antibodies against *S.* Enteritidis C50041 OMPs in the serum and intestinal mucosal lavage fluid of immunized chickens via ELISA. Specific anti-C50041 OMPs antibodies were induced in all immunized groups, including the rSC0169 (cultured with or without rhamnose) and the wild-type *S.* Enteritidis C50041 groups. Notably, compared with the rSC0169 group (cultured without rhamnose) and wild-type *S.* Enteritidis C50041 group, the rSC0169 (+rhamnose) group induced significantly higher IgY (1.43-fold and 1.66-fold, respectively) and IgA (2.05-fold and 2.45-fold, respectively) antibody titers (*p* < 0.05; Figure 2D). These findings demonstrate that the rhamnose-regulated delayed attenuation system enhances the immunogenicity of the *S.* Enteritidis vector rSC0169.

### 3.6. Construction, Growth Curve, and Genetic Stability of rSC0169(pS-EntB)

**Figure 3:**
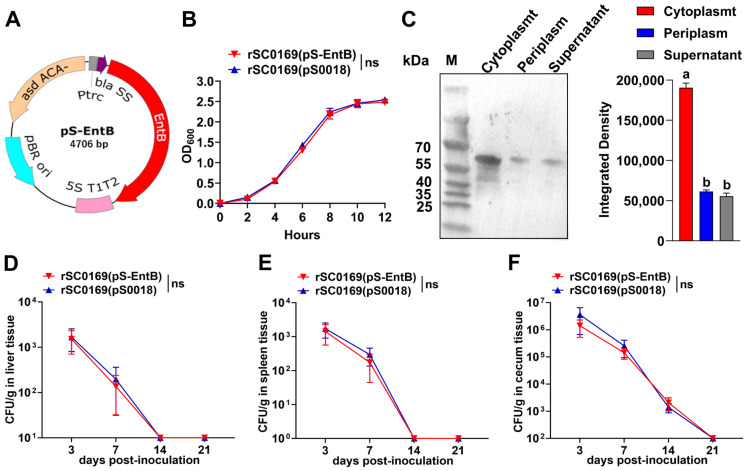
Phenotypic identification of the recombinant strain rSC0169(pS-EntB). (**A**) Plasmid map of the recombinant plasmid pS-EntB. (**B**) Growth curves of the control strain rSC0169(pS0018) and the recombinant strain rSC0169(pS-EntB). (**C**) Subcellular localization of EntB in the recombinant strain rSC0169(pS-EntB). Distinct letters indicate significant differences between groups (*p* < 0.05). (**D**–**F**) Bacterial loads (CFU/g) in chicken liver (**D**), spleen (**E**), and cecum (**F**) at 3, 7, 14, and 21 dpi with rSC0169(pS0018) or rSC0169(pS-EntB). Data are mean ± SD (*n* = 3). ns means not significant. Original images of (**C**) can be found in Supplementary Materials.

The recombinant plasmid pS-EntB was electrotransformed into strain rSC0169 to successfully construct the candidate vaccine rSC0169(pS-EntB) (Figure 3A). Heterologous antigen delivery can impair vector growth and reduce vector antigen immunogenicity [25]. To assess whether EntB affects the growth of the *Salmonella* vector, we compared the growth characteristics of rSC0169(pS-EntB) with the empty vector strain rSC00169(pS0018). No significant growth difference was observed between the two strains, indicating that EntB antigen does not impose metabolic burden or growth inhibition on the vector (Figure 3B). To evaluate the genetic stability of the recombinant plasmid pS-EntB in rSC0169, the strain was serially passaged for 50 generations. PCR verification confirmed the presence of pS-EntB in all tested colonies, indicating excellent genetic stability of the recombinant plasmid in this *Salmonella* vector.

### 3.7. EntB Protein Expression in rSC0169(pS-EntB) Subcellular Fractions

Heterologous antigen expression and secretion by *Salmonella* vectors are prerequisites for inducing specific immune responses [15]. To assess EntB expression in rSC0169(pS-EntB), Western blot analysis was conducted with anti-EntB polyclonal antiserum. Specific immunoreactive bands were detected in the cytoplasmic, periplasmic, and culture supernatant fractions of rSC0169(pS-EntB) (Figure 3C). These data confirm that EntB is synthesized in rSC0169 and simultaneously secreted into the periplasm and extracellular space.

### 3.8. Tissue Colonization of rSC0169(pS-EntB)

Colonization capacity reflects the live vector-host lymphoid tissue interaction and strongly correlates with vector immunogenicity [8]. To evaluate tissue colonization dynamics of *Salmonella*, chickens were orally administered nalidixic acid-resistant mutants rSC0169(pS0018) and rSC0169(pS-EntB). Bacterial loads in liver, spleen, and cecum tissues were quantified at 3, 7, 14, and 21 dpi. Both strains effectively colonized the liver, spleen, and cecum tissues of chickens at 3 and 7 dpi (Figure 3D–F), but were fully cleared from the liver and spleen by 14 dpi (Figure 3D,E) and from the cecum by 21 dpi (Figure 3F). Collectively, this colonization profile ensures the vector has sufficient time to effectively stimulate the host lymphatic system, and the subsequent elimination guarantees the biosafety in the later stage.

### 3.9. rSC0169(pS-EntB) Induces Robust Humoral and Mucosal Immune Responses in Chickens

**Figure 4:**
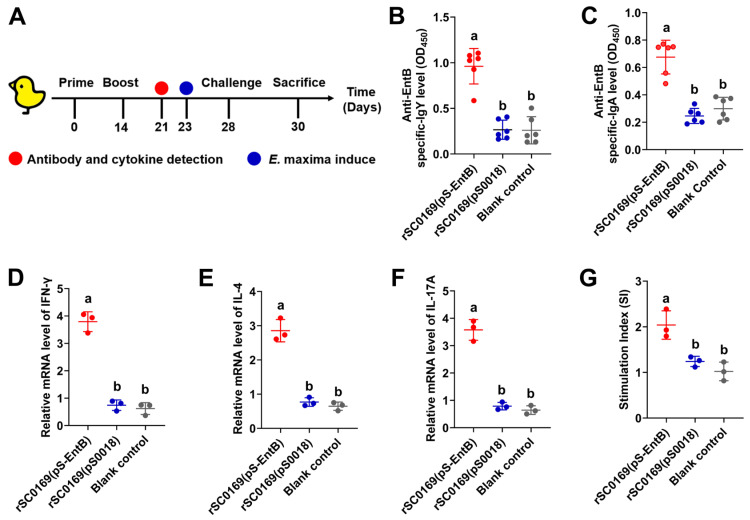
Immune responses induced in chickens immunized with rSC0169(pS-EntB). (**A**) Schematic diagram of the chicken immunization and challenge experiment. (**B**,**C**) Specific anti-EntB IgY (**B**) and IgA (**C**) titers in chicken serum and intestinal mucosal rinse measured by ELISA. Data are mean ± SD (*n* = 6). (**D**–**G**) mRNA expression levels of IFN-γ (**D**), IL-4 (**E**), and IL-17A (**F**) and proliferation response (**G**) in peripheral blood lymphocytes stimulated with EntB protein. Data are presented as mean ± SD (*n* = 3). Distinct letters indicate significant differences between groups (*p* < 0.05).

To evaluate the humoral and mucosal immune responses, anti-EntB IgY and IgA titers were measured (Figure 4A). Chickens orally immunized with rSC0169(pS-EntB) exhibited significantly higher anti-EntB IgY (3.63-fold; *p* < 0.001) and IgA (2.75-fold; *p* < 0.001) antibody levels than those in the rSC0169(pS0018) group (Figure 4B,C). These data confirm that rSC0169(pS-EntB) elicits robust humoral and mucosal immunity in chickens.

### 3.10. rSC0169(pS-EntB) Induces Strong Cellular Immune Responses in Chickens

To assess the cellular immune responses, cytokine production and PBMCs proliferation assays were conducted (Figure 4A). PBMCs isolated from the rSC0169(pS-EntB)-immunized group produced significantly elevated levels of IFN-γ (5.10-fold; *p* < 0.001), IL-4 (3.71-fold; *p* < 0.001), and IL-17A (4.52-fold; *p* < 0.001) cytokines following stimulation with EntB, compared with those from the rSC0169(pS0018) group (Figure 4D–F). Consistently, PBMC proliferation activity was markedly elevated in the rSC0169(pS-EntB) group relative to the rSC0169(pS0018) group (1.64-fold; *p* < 0.05; Figure 4G). These data demonstrate that rSC0169(pS-EntB) elicits robust cellular immunity in chickens.

### 3.11. rSC0169(pS-EntB) Alleviates Small Intestinal Injury Caused by C. perfringens in Chickens

**Figure 5:**
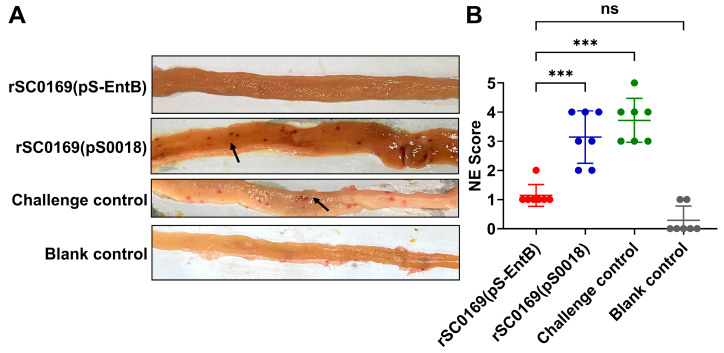
Intestinal lesion score in chickens challenged with *C. perfringens*. Chickens were euthanized and dissected at 4 h post-challenge, and scoring was performed in a blinded manner. (**A**) Representative images of small intestinal lesions. Black arrows indicate punctate necrosis. (**B**) NE lesion scores. Data are mean ± SD (*n* = 7). ns means not significant. ***, *p* < 0.001.

**Figure 6:**
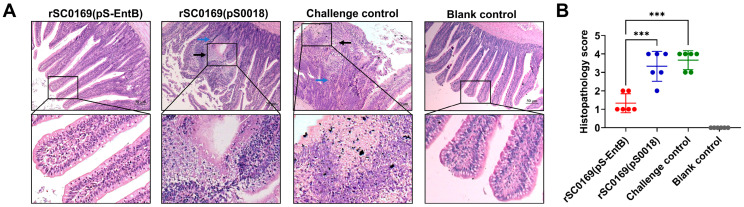
Histopathological examination of small intestine in chickens challenged with *C. perfringens*. Chickens were euthanized at 4 h post-challenge, and intestinal tissues were stained with H&E. Scoring was performed in a blinded manner. (**A**) Representative histopathological images of small intestinal tissue. Lymphocyte infiltration is indicated by the blue arrows, and villous shedding by black arrows. Scale bar in the upper panel: 50 μm. The lower panel presents a 4× magnification view of the boxed area in the upper panel, with the magnification factor determined by the proportional increase in both the height and width of the framed region. (**B**) Histopathological lesion scores. Data are mean ± SD (*n* = 6). ***, *p* < 0.001.

Intestinal mucosal injury is a typical pathological hallmark of NE [26]. To evaluate the protective efficacy of rSC0169(pS-EntB), intestinal lesion scoring was performed (Figure 4A). No necrotic lesions were observed in the blank control or coccidia control groups, whereas extensive punctate necrosis was observed in the challenge and rSC0169(pS0018) groups (Figure 5A). The rSC0169(pS-EntB)-immunized group exhibited markedly reduced lesion scores than the rSC0169(pS0018) group (*p* < 0.001), with no significant difference relative to the blank control group (Figure 5B). Consistently, small intestinal histopathology revealed that the challenge and rSC0169(pS0018) groups exhibited obvious lymphocyte infiltration (blue arrows), villous necrosis, and dissolution (black arrows) compared with normal tissues. In contrast, the rSC0169(pS-EntB)-immunized group showed relatively intact mucosal structures, minimal epithelial shedding, and mild lymphocyte infiltration (Figure 6A). Correspondingly, the histopathological scores in the rSC0169(pS-EntB) group were markedly lower than in the rSC0169(pS0018) group (*p* < 0.001; Figure 6B). The results indicated that rSC0169(pS-EntB) effectively alleviates small intestinal injury in chickens.

### 3.12. rSC0169(pS-EntB) Improves the Morphology of Small Intestinal Villi in Chickens

**Figure 7:**
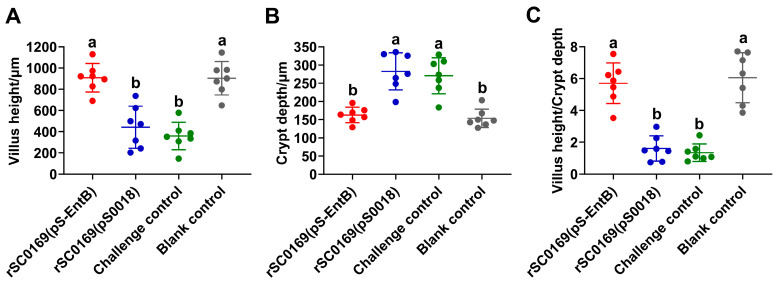
Effect of candidate vaccine on small intestinal morphology in chickens challenged with *C. perfringens*. Intestinal morphology was measured at 4 h post-challenge. Five intact villi per tissue section were randomly selected for measurement. (**A**) Villi height. (**B**) Crypt depth. (**C**) Villi height to crypt depth ratio. Data are mean ± SD (*n* = 7). Distinct letters indicate significant differences between groups (*p* < 0.05).

The VH/CD is a key morphological indicator of small intestinal digestive and absorptive function [27]. To assess the protective effect of rSC0169(pS-EntB) on intestinal mucosal morphology, small intestinal tissue sections were prepared. Relative to the blank control group, the challenge and rSC0169(pS0018) groups exhibited significantly reduced villus height (*p* < 0.001) and VH/CD ratio (*p* < 0.001), and increased crypt depth (*p* < 0.001) (Figure 7A–C). Conversely, chickens immunized with rSC0169(pS-EntB) showed significantly higher villus height (2.06-fold; *p* < 0.001) and VH/CD (3.45-fold; *p* < 0.001), and lower crypt depth (0.57-fold; *p* < 0.001), compared with rSC0169(pS0018) group (Figure 7A–C), with no statistical difference from the blank control. These results indicate that rSC0169(pS-EntB) can effectively improve the morphological structure of the intestinal villi, thereby facilitating the restoration of the digestive and absorptive functions of the intestine.

### 3.13. rSC0169(pS-EntB) Significantly Upregulates Intestinal Tight Junction Proteins and Reduces Intestinal Inflammation in Chickens

**Figure 8:**
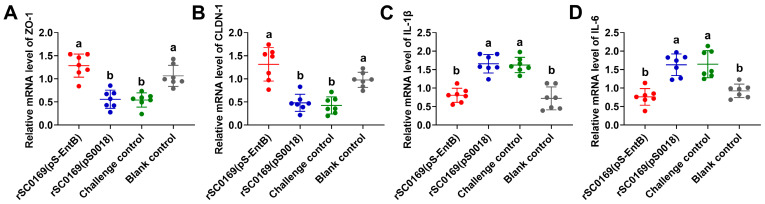
Effect of candidate vaccine on intestinal tight junction proteins and pro-inflammatory cytokine expression in chickens challenged with *C. perfringens*. Total RNA was extracted from small intestinal tissues at 4 h post-challenge and analyzed by qRT-PCR. Relative gene expression was calculated using the 2^−ΔΔCt^ method with β-actin as the reference gene. (**A**–**D**) Expression levels of tight junction proteins ZO-1 (**A**), CLDN-1 (**B**), and pro-inflammatory cytokines IL-1β (**C**) and IL-6 (**D**). Data are mean ± SD (*n* = 7). Distinct letters indicate significant differences between groups (*p* < 0.05).

Intestinal barrier integrity and the inflammatory status are core indicators of intestinal health [28,29]. To clarify the underlying mechanism of intestinal protection, total RNA was isolated from small intestine tissues, and qRT-PCR was used to detect mRNA expression of ZO-1, CLDN-1, IL-1β, and IL-6. Relative to the blank control group, the challenge and rSC0169(pS0018) group exhibited significantly downregulated mRNA expression of ZO-1 (*p* < 0.001) and CLDN-1 (*p* < 0.01). In contrast, the rSC0169(pS-EntB)-immunized group showed markedly increased expression levels of ZO-1 (2.30-fold; *p* < 0.001) and CLDN-1 (2.73-fold; *p* < 0.001) relative to the rSC0169(pS0018) group, with no significant difference from the blank control group (Figure 8A,B). Correspondingly, IL-1β (*p* < 0.001) and IL-6 (*p* < 0.001) mRNA transcription was significantly upregulated in the challenge and rSC0169(pS0018) groups relative to the blank control, whereas IL-1β (0.49-fold; *p* < 0.001) and IL-6 (0.47-fold; *p* < 0.001) expression was significantly suppressed in the rSC0169(pS-EntB)-immunized group, which showed no difference from the blank control group (Figure 8C,D). These results indicated that rSC0169(pS-EntB) enhances intestinal barrier function and alleviates intestinal inflammatory responses.

## 4. Discussion

*C. perfringens* exotoxins are largely responsible for NE pathogenesis. EntB was predicted as a putative enterotoxin [1]. Therefore, characterizing its structural features is of great theoretical value for NE vaccine development. Bioinformatic analysis showed that EntB comprises two domains: the NlpC and YgiM domains (Figure 1D). Given that the NlpC domain typically exhibits hydrolase activity, we hypothesized that this domain may disrupt the intestinal barrier to mediate NE pathology [30]. The YgiM domain, as a membrane-related domain, may promote EntB adhesion to host intestinal epithelial cells [31]. Collectively, these predicted structural characteristics support EntB as a promising antigen target for necrotic enteritis vaccine development. To our knowledge, this study represents the first attempt to deliver EntB using an attenuated *Salmonella* vector with a rhamnose-regulated delayed attenuation system, which confers novelty to the present work.

Balancing immunogenicity and biosafety represents a key challenge for attenuated *Salmonella* vectors, as excessive attenuation often compromises immunogenicity and may result in inadequate protection [7]. The rhamnose-regulated delayed attenuation system can simultaneously enhance the immunogenicity and biosafety of *Salmonella* vectors [10]. In this study, the rhamnose-dependent promoter in rSC0169 remains inactive in the chicken host due to the absence of rhamnose, locking the strain in a rough LPS phenotype with reduced inflammatory potential. Correspondingly, the rSC0169 exhibited significantly reduced pro-inflammatory cytokine levels and milder histopathological lesions in the liver compared with wild-type *S.* Enteritidis C50041 (Figure 2A,B). This in vivo phenotypic switch explains the improved safety profile, avoiding excessive tissue damage while preserving colonization capacity. These observations are consistent with previous studies demonstrating that regulated delayed attenuation improves the safety of *Salmonella* vector vaccines without impairing colonization efficiency [32]. These findings further confirmed that the rhamnose-regulated delayed attenuation system enables rSC0169 to achieve effective attenuation in the host, thus enhancing its biosafety and providing a solid foundation for its development as a live vector vaccine.

On the basis of the established biosafety of rSC0169, we further evaluated its immunogenicity to determine whether the delayed attenuation system preserves the immunostimulatory capacity of the *S.* Enteritidis vector. In vitro rhamnose induction enabled rSC0169 to activate the IRF3-dependent innate immune pathway (Figure 2C), indicating that the vector maintained a structurally intact LPS profile under in vitro induction. A stable LPS structure is essential for the maturation of antigen-presenting cells (APCs) and the efficient initiation of adaptive immune responses [33]. Notably, rSC0169 cultured with rhamnose elicited significantly higher OMPs-specific antibody titers than the vector cultured in the absence of rhamnose (Figure 2D), supporting that the system enhances the vector’s immunogenicity. Mechanistically, the rhamnose-regulated delayed attenuation system drives a time-dependent smooth-to-rough LPS phenotypic switch in vivo. During early intestinal colonization, the vector exhibits a smooth LPS phenotype to ensure efficient invasion and persistence in host lymphoid tissues. As delayed attenuation proceeds, the vector gradually shifts to a rough LPS phenotype, which exposes OMPs and supports sustained release and presentation of the heterologous antigen EntB. This kinetically optimized antigen exposure enhances APC activation, boosts immune responses against OMPs, and avoids excessive toxicity, thereby reconciling immunogenicity and biosafety. This favorable antigen exposure pattern also provides a solid basis for improving the immunogenicity of the heterologous antigen EntB delivered by the rSC0169 vector. Furthermore, rSC0169(pS-EntB) induced markedly stronger mucosal immune responses than control vector rSC0169(pS0018) (Figure 4C,D,F). Mucosal immunity effectively inhibits the early adhesion and invasion of *C. perfringens* on the intestinal epithelial surface, which plays a pivotal role in NE prevention [34]. This finding aligns with numerous studies highlighting mucosal immune activation as a key defense mechanism against necrotic enteritis [35]. Collectively, these results demonstrate that the rhamnose-regulated delayed attenuation system preserves the immunostimulatory capacity of the *S.* Enteritidis vector while ensuring its biosafety. This finding provides novel insights into addressing the long-standing dilemma in attenuated *Salmonella* vectors.

*C. perfringens* infection disrupts intestinal epithelial integrity, impairs physical barrier function, and ultimately triggers excessive inflammation [28,36]. Intestinal barrier function largely depends on intact tight junction complexes, which are critical for maintaining epithelial permeability and preventing pathogen invasion and uncontrolled inflammatory infiltration [37]. Inflammatory signaling activated by *C. perfringens* further damages tight junctions, forming a vicious cycle that exacerbates barrier dysfunction and tissue injury [38,39]. Therefore, vaccines targeting NE are crucial for repairing barrier function and reducing inflammatory responses. In this study, rSC0169(pS-EntB) increased the VH/CD ratio (Figure 7C), significantly upregulated the expression of intestinal tight junction proteins, and markedly reduced the expression of pro-inflammatory cytokines (Figure 8A–D). Specifically, the enhanced expression of tight junction proteins contributed to maintaining intestinal structural integrity and limiting excessive inflammatory responses, thereby alleviating intestinal damage caused by *C. perfringens* infection [37,40]. Collectively, these results suggest that EntB delivered by the *Salmonella* vector rSC0169 can effectively maintain intestinal morphological integrity in chickens infected with *C. perfringens* by strengthening the intestinal barrier and mitigating excessive inflammation. This discovery further demonstrates the potential of EntB as a key protective antigen against NE and verifies that rSC0169 serves as an efficient antigen delivery platform, which can effectively enhance the capacity of EntB to promote intestinal barrier repair and resist intestinal pathogenic bacterial invasion. Notably, most existing *Salmonella*-vectored vaccines against *C*. *perfringens* primarily focus on evaluating immune responses and protective efficacy, while few studies have assessed intestinal barrier integrity, villus morphology, or inflammatory cytokine profiles. In contrast to these previous reports, our study provides a more comprehensive assessment of intestinal protection [10,41,42]. Our findings, therefore, provide a theoretical basis for the design of mucosal vaccines targeting intestinal barrier repair and inflammatory regulation in poultry.

Finally, genetic modifications further enhanced the biosafety of the rSC0169 vector and reduced the risks of environmental transmission and horizontal gene transfer. These modifications included: the Δ*asdA* balanced-lethal system to restrict environmental growth, Δ*recF* to reduce homologous recombination, Δ*sifA* to promote immune clearance, and the SIRV system for programmed lysis. In vivo experimental results confirmed the favorable safety profile of rSC0169, with reduced inflammation and milder pathological lesions. The vector contains no antibiotic resistance genes or mobile genetic elements, and strict biosafety measures have been implemented to prevent environmental release. In summary, these characteristics demonstrate that rSC0169 is a safe, efficient, and low-risk vector for delivering protective antigens against necrotic enteritis in chickens.

## 5. Conclusions

In summary, our findings demonstrate that the *S.* Enteritidis vector rSC0169, which harbors the rhamnose-regulated delayed attenuation system, effectively enhances the immunogenicity of EntB, and the recombinant strain rSC0169(pS-EntB) confers effective protection against NE by maintaining the intestinal barrier function and reducing the inflammatory response. These findings provide a promising NE candidate vaccine and offer a valuable strategy for developing vaccines against other intestinal bacterial diseases. Nevertheless, some limitations of this study should be acknowledged. First, the necrotic enteritis challenge model used in this study may not fully reflect field conditions. Second, the sample size was relatively small; studies with larger populations are needed to verify the protective efficacy. Further research will also focus on evaluating long-term immune persistence and practical field application of this vaccine.

## Figures and Tables

**Table 1:** Bacterial strains and plasmids.

Strains and Plasmids	Characteristics ^a^	Source or Reference
Bacterial strains		
BL21	For expression of the recombinant plasmids	Invitrogen (Carlsbad, CA, USA)
χ7213	*thi-1 thr-1 leuB6 fhuA21 lacY1 glnV44 asdA4 recA1 RP4 2-Tc::Mu pir*; *Km^r^*	Lab stock
rSC0163	Δ*relA*::*araC* P_araBAD_ *lacI* TT Δ*endA*::*araC* P_araBAD_ *mazE* TT Δ*manA* Δ*cysG*:P_lac_ *mazF*	[12]
rSC0169	Δ*relA*::*araC* P_araBAD_ *lacI* TT Δ*endA*::*araC* P_araBAD_ *mazE* TT Δ*manA* Δ*cysG*:P_lac_ *mazF* Δ*sifA* Δ*waaL* Δ*pagL64*::TT *rhaRs*P_rhaBAD_ *waaL* Δ*recF* Δ*asdA*	This study
rSC0097	*Clostridium perfringens* Type G, Wild type, α + NetB +	Lab stock
rSC0169(pS0018)	A recombinant *S.* Enteritidis vector containing plasmid pS0018	Lab stock
rSC0169(pS-EntB)	A recombinant *S.* Enteritidis vector containing plasmid pS-EntB	This study
Plasmids		
pRE112	*oriT oriV sacB Cm^r^*	Lab stock
∆*sifA*	Suicide vector for Δ*sifA*, pRE112	[8]
Δ*waaL*	Suicide vector for Δ*waaL*, pRE112	
∆*pagL*64::TT *rhaRS* PrhaBAD *waaL*	Suicide vector for Δ*pagL*64::TT *rhaRS* PrhaBAD *waaL*, pRE112	
Δ*recF*	Suicide vector for Δ*recF*, pRE112	Lab stock
Δ*asdA*	Suicide vector for Δ*asdA*, pRE112	[8]
pS0018	A derivative of plasmid pYA3493 has removed the ACA sequence from the asd gene without altering the amino acid sequence. Asd^+^; pBR *ori*, P_trc_ promoter, β-lactamase signal sequence-based periplasmic secretion plasmid	[9]
pS-EntB	pS0018 with EntB, P_trc_ promoter	Lab stock
pET28a(+)-EntB	A recombinant expression vector containing EntB; *Km^r^*	[1]

^a^ *Km^r^* Kanamycin resistance. *Cm^r^* Chloramphenicol resistance.

**Table 2:** The primer information.

Primers Name	Sequences (5′-3′) Forward Primer/Reverse Primer	Reference
∆*sifA*	TGATGAGCTCTTTCTCTTCTCCAAAATCTC/CTTAGGTACCGGTCGATTTAATCAATTATG	[8]
Δ*waaL*	GCTGCTCACCAGAACAGAAC/AATGTAGCGCAGCGTTTC	
∆*pagL*64::TT *rhaRS* P_rhaBAD_ *waaL*	GGGATTATGCCATTCATAAGCT/ATGCCTGCACTAAACCAGATG	
Δ*recF*	ATCGGCTTTAACGTCAGTTACGT/GCCGCCGAGACGCCTTCTTCCGG	
Δ*asdA*	TGCTCTAGATGTGCATGGCAATCGCCCAAC/TCCCCCGGGTATCTGCGTCGTCCTACCTTC	
pS0018	AACGCTGGTGAAAGTAAAAGATG/CAGACCGCTTCTGCGTTCT	[9]
IL-4	TGAATGACATCCAGGGAGAG/GGCTTTGCATAAGAGCTCAG	[14]
IFN-γ	AGCTGACGGTGGACCTATTATT/GGCTTTGCGCTGGATTC	
IL-17A	CTCCGATCCCTTATTCTCCTC/AAGCGGTTGTGGTCCTCAT	
ZO-1	GGATGTTTATTTGGGCGGCT/CCATTGTTGCACTCTTGCCG	[17]
Claudin-1	CACACCCGTTAACACCAGATTT/GAGGGGGCATTTTTGGGGTA	
IL-1β	GTACCGAGTACAACCCCTGC/AGCAACGGGACGGTAATGAA	[18]
IL-6	CAAGGTGACGGAGGAGGAC/TGGCGAGGAGGGATTTCT	
IL-18	GGAATGCGATGCCTTTTG/ATTTTCCCATGCTCTTTCTCA	[13]
β-actin	CAACACAGTGCTGTCTGGTGG/ATCGTACTCCTGCTTGCTGATCC	[14]

## Data Availability

The materials and data not presented in this manuscript are available from the corresponding author upon request.

## Supplementary Materials

The following supporting information can be downloaded at: https://www.mdpi.com/article/10.3390/biom16040575/s1, Figure S1. Original images of Figure 2C. Figure S2. Original images of Figure 3C.
