# Supplementary figures and images for: Recombinant Attenuated Salmonella Enteritidis Vector Enhances the Immunogenicity of Clostridium perfringens EntB Antigen for Effective Prevention of Avian Necrotic Enteritis
Source: Biomolecules. 2026 Apr 13;16(4):575. doi: 10.3390/biom16040575 (PMC13115302; doi:10.3390/biom16040575)

## Supplementary figure

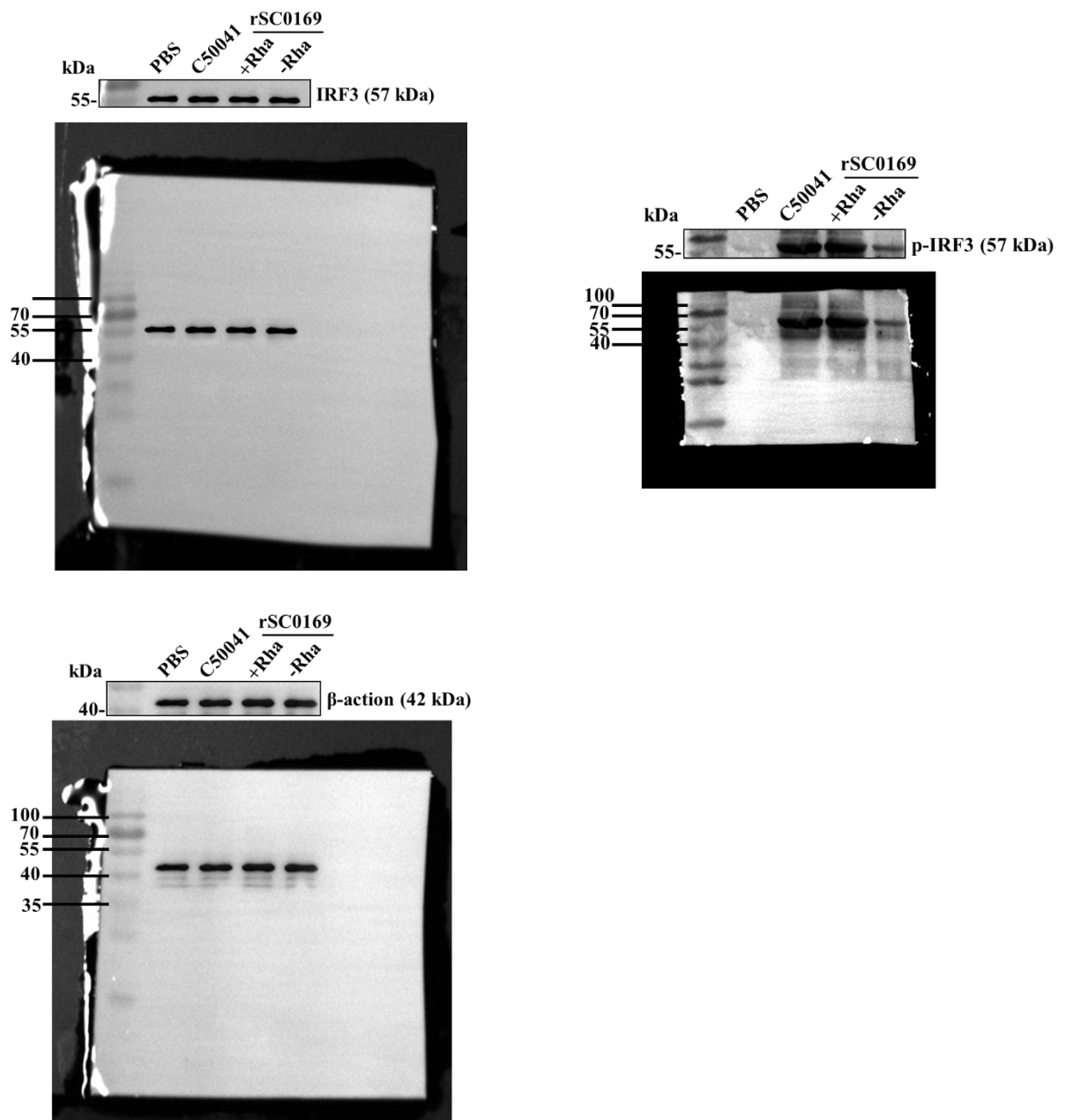

Figure S1. Original images of figure 2C.

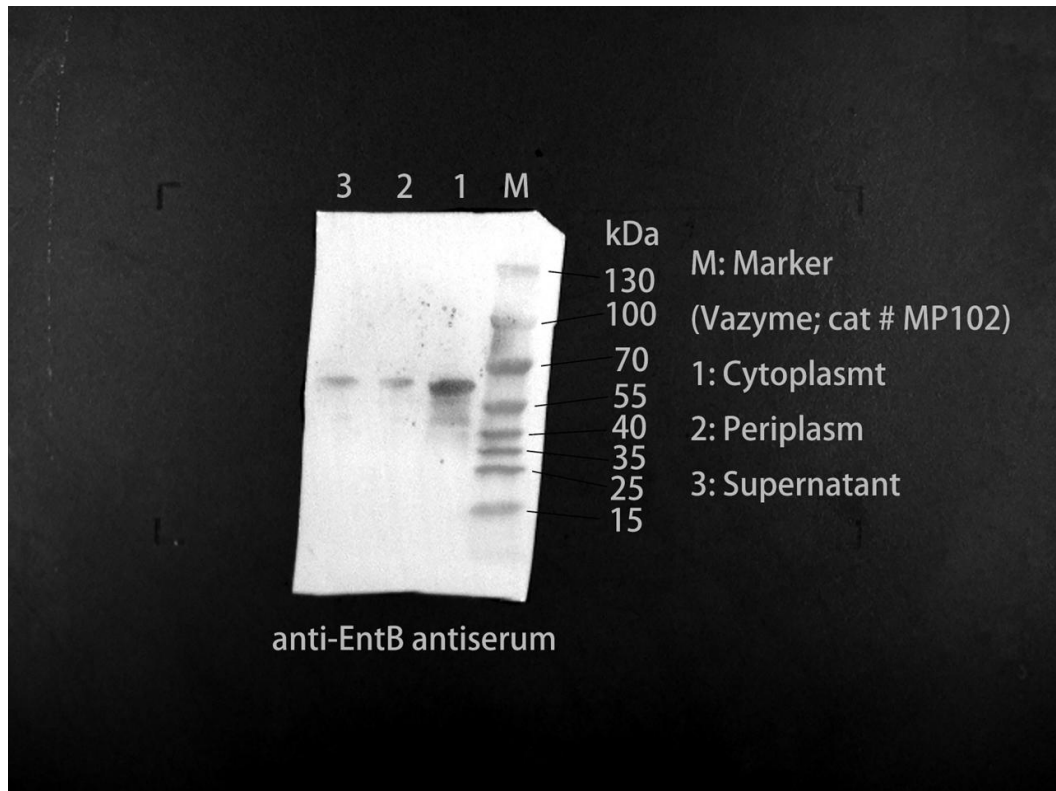

Figure S2. Original images of figure 3C.

Supplement: Supplementary file 1 [file biomolecules-16-00575-s001.zip › biomolecules-4197170-supplementary.pdf]
